# Analysis of the diversity and function of the microbiota in infected root canals of type 2 diabetes mellitus with apical periodontitis

**DOI:** 10.3389/fcimb.2025.1514510

**Published:** 2025-05-08

**Authors:** Yuzhi Li, Song Wu, Yong Zhang, Ting Zhang, Can Hu, Peng Zhang, Wanrong Yang, Sheng Xiong, Yanni Liu

**Affiliations:** ^1^ Department of Stomatology, Guang’an People’s Hospital, Guang’an, Sichuan, China; ^2^ First Clinical Division, Peking University School and Hospital of Stomatology & National Center of Stomatology & National Clinical Research Center for Oral Diseases & National Engineering Laboratory for Digital and Material Technology of Stomatology & Beijing Key Laboratory of Digital Stomatology, Beijing, China

**Keywords:** apical periodontitis, type 2 diabetes mellitus, root canal, microbiome, sequencing

## Abstract

**Purpose:**

This study aimed to compare the compositional differences and functions of microbial communities in infected root canals of teeth with apical periodontitis (AP) and/or type 2 diabetes mellitus (T2DM) using 16S rDNA sequencing.

**Methods:**

Eighteen participants were categorized into two groups based on their health conditions: AP and AP complicated with T2DM (APDM). Infected root canal microbiota was clinically collected for 16S rDNA sequencing. Subsequent statistical and bioinformatics analyses were conducted after sequencing by NovaSeq, encompassing diversity analysis, intergroup difference analysis, and functional prediction analysis.

**Results:**

The core microbiome of root canal microorganisms was similar in the two groups, which mainly consisted of *Bacteroidota, Firmicutes, Synergistota, Actinobacteriota, Proteobacteria, Fusobacteriota, Spirochaetota*. The root canals of the affected teeth of APDM had significantly higher abundance of *Olsenella_uli, Peptostreptococcaceae_bacterium _oral_taxon_113_str_W5053, W5053, Pyramidobacter_piscolen, Pyramidobacte, Synergistaceae, Synergistales* was significantly higher than AP group (P<0.05). Under the condition of T2DM, root canal microbial metabolism was predominantly enriched towards functions of the phosphotransferase system, ABC system, amino acid metabolism, and carbohydrate metabolism.

**Conclusion:**

The microbial community in the infected root canals of APDM showed similarities to AP yet exhibited differences in certain species and microbial functions.

## Introduction

Apical periodontitis (AP), a prevalent endodontic disease, is primarily triggered by bacterial infections and often stems from dental caries, pulp necrosis, or periodontitis. Epidemiological surveys estimate that approximately 52% of adults globally have at least one tooth affected by AP (Tibúrcio-MaChado et al., 2021). Recent research indicates that AP not only results in local periapical tissue inflammation and bone resorption but also correlates with systemic factors such as diabetes, hyperlipidemia, and smoking, potentially influencing the patient’s overall health (Liu et al., 2023a; Vasques et al., 2023; Xiao et al., 2023).

Diabetes mellitus, a collection of endocrine and metabolic disorders marked by hyperglycemia due to insulin secretion defects or insulin resistance (Heald et al., 2020), affects a significant portion of the global population. The International Diabetes Federation (IDF) reports that the number of individuals with diabetes reached 537 million in 2021, with projections nearing 700 million by 2045 (Sun et al., 2022). Type 2 diabetes mellitus (T2DM), predominantly caused by insulin resistance and often due to insufficient insulin secretion from pancreatic β-cell deterioration, is most common in individuals over 40 and accounts for over 90% of diabetes cases (Ahmad et al., 2022). Studies have shown that diabetes can lead to systemic chronic inflammation, impair immune cell function, and cause organ and tissue damage, suggesting a close link between T2DM and AP development (Ye et al., 2023). A recent study found that patients with T2DM have nearly three times the risk of developing periapical infections compared to non-diabetic patients (Liu et al., 2023b). Moreover, those with T2DM-AP undergo longer and less successful root canal treatments and are more prone to persistent periapical infections (Jakovljevic and Duncan, 2020; Martinho et al., 2021). The healing of periapical lesions in T2DM patients is also significantly delayed, sometimes extending to 4 years (Smadi, 2017). These findings imply that hyperglycemia in diabetic patients affects the onset, progression, and prognosis of AP, although the precise mechanisms are not yet fully understood.

Bacteria may significantly influence the development of both AP and T2DM. The absence of an epithelial barrier between the infected root canal and the periapical blood and lymphoid tissue facilitates the entry of pathogenic microorganisms into the bloodstream, increasing the risk of bacteremia and potentially impacting host glucose metabolism (Bordagaray et al., 2021). Additionally, DM has been shown to enhance the pathogenicity of the oral microbiome by inducing systemic inflammation (Saeb et al., 2019). Studies have reported a significant increase in Gram-negative obligate anaerobic bacteria in the root canals of diabetic rats, suggesting that metabolic disruptions associated with DM could weaken the host’s defense against microbial infections (Iwama et al., 2006).

Previous investigations into the bacterial distribution within infected root canals of individuals with T2DM and AP have relied on traditional methods such as bacterial culture identification and PCR, which have limitations in precision and coverage. This has led to a paucity of robust studies on the microorganisms associated with infected root canals in the context of T2DM. The advent of 16S rDNA sequencing has provided a powerful tool for exploring microbial diversity and for a more comprehensive analysis of the structural composition and function of microbial communities (Zheng et al., 2022).

In this study, we employed 16S rDNA sequencing to examine the microbial presence in infected root canals associated with AP, in the presence and absence of T2DM. Our aim was to compare the diversity and functionality of microbial communities under these two conditions and to provide further evidence of the impact of T2DM on periapical disease pathogenesis.

## Materials and methods

### Participant recruitment

This study received approval from the Ethics Committee of Guang’an People’s Hospital, Guang’an, Sichuan, China (ethics approval number: KY202305243). Written informed consent was obtained from all participants prior to their involvement. The clinical procedures adhered strictly to the Declaration of Helsinki and the Good Clinical Practice Guidelines. Upon completion of sampling, all participants were offered a comprehensive root canal treatment at no cost.

The sample size of this study was established with guidance from a qualified biostatistician. Three studies that used a similar NGS approach in the form of pyrosequencing reported the recruitment of 10, 12, and 21 participants, respectively (Hou et al., 2022; Lu et al., 2022; Pérez-Carrasco et al., 2023). The typical pilot study utilizing a sample size of 30 is not relevant in this case, as the outcome we observed was anticipated to deviate from a normal distribution. Therefore, based on this and previous literature as well as the time available for patient recruitment and sampling, which was limited, we proposed the recruitment of 20 participants with an expected dropout rate of,15%.

### Patients

AP was diagnosed by seasoned clinicians in accordance with the clinical practice guideline for pulpal and apical diseases published by the American Academy of Endodontists in 2014. T2DM diagnosis followed the criteria outlined by the American Diabetes Association in 2014. Participants were recruited from the outpatient department of the Department of Stomatology at Guang’an People’s Hospital, Guang’an, Sichuan, China.

Participants were included and divided into 2 groups according to detailed inclusion criteria, as follows:

- The AP group comprised patients with apical periodontitis:
  - HbA1c levels were below 6.5%;
  - Patients tested negative on sensibility tests;
  -Diseased teeth exhibited AP lesions as confirmed radiographically.
- The APDM group included subjects with apical periodontitis complicated by T2DM:
  - Participants had been diagnosed with T2DM at least 2 years prior;
  - Random blood sugar levels or 2-hour oral glucose tolerance test (OGTT) results exceeded 11.1 mmol/L;
  - HbA1c levels, regularly monitored over the 2 years preceding our study, were above 7%;
  - Patients tested negative on sensibility tests;
  -Diseased teeth exhibited AP lesions as confirmed radiographically.
- Participants were excluded based on the following exclusion criteria:
  - Presence of systemic diseases other than T2DM;
  - Prior root canal treatment;
  - Apical lesions directly communicating with the oral cavity due to periodontitis;
  - Antibiotic treatment within the last 3 months;
  - History of smoking;
  - Pregnancy.

### Sample collection

Participants were instructed to rinse with a 0.2% chlorhexidine solution for 3 minutes. After disinfecting the affected tooth and surrounding area with a 1% iodine tincture, a rubber dam was used to isolate the affected teeth. A sterile high-speed handpiece and carbide burs cooled with sterile water were used to remove enamel, dentin, or any residual filling material from the affected tooth. A sterile round bur was employed to create an anhydrous environment while accessing the pulp chamber. The root canals were examined with a sterile probe and purged with a size 15-K file. The working length was established using an electronic apex locator, and the root canal walls were filed repeatedly. After preparation, the root canals were dried by absorbing fluids with a sterile paper point for 30 seconds. Then, 200 μL of sterile saline solution was introduced into the root canal, collected with another sterile paper point, and this process was repeated once more. The three collection paper points, along with the size 15-K file tip, were collected and placed into a 1.5 mL centrifuge tube containing 1 mL of TE buffer. The tube was labeled, gently mixed, and stored at -80°C (Nardello et al., 2020; Lopes et al., 2021).

### DNA extraction and 16S rDNA sequencing

Samples were processed using the QIAamp DNA extraction kits (Qiagen, Hilden, Germany) as per the manufacturer’s instructions. Total DNA was extracted and stored at -20°C for further analysis. DNA quantities and qualities were assessed using a NanoDrop ND-2000 spectrophotometer (Thermo Fisher Scientific, Waltham, MA, USA) and agarose gel electrophoresis, respectively. PCR amplifications targeted the variable V3-V4 regions of the 16S rDNA gene with bacterial domain-specific primers (341F: 5’-CCTACGGGNGGCWGCAG-3’ and 805R: 5’-GACTACHVGGGTATCTAATCC-3’). After amplifying the bacterial genomic DNA, a library was constructed using a Library Prep Kit (Thermo Fisher, Waltham, MA, USA) according to the manufacturer’s protocol. The library was validated using an Agilent 2100 Bioanalyzer (Agilent, Santa Clara, CA, USA) and sequenced on an Illumina NovaSeq platform (NovaSeq6000 PE250) (Illumina, San Diego, CA, USA).

### Sequence analysis

High-quality data were subjected to comprehensive bioinformatic analyses to assess the root canal plaque samples. Alpha/beta diversity analysis was conducted to evaluate the microbiome diversity within each sample and species composition variation among samples. The Shannon/Chao1 index was applied to measure species diversity and abundance, while analysis of similarity was used to evaluate compositional heterogeneity. Microbial species abundance in the root canal was compared across different groups. LEfSe analysis was also performed to identify key bacterial taxa contributing to disease development. Additionally, Kyoto Encyclopedia of Genes and Genomes (KEGG) functional enrichment analysis was conducted to compare functional pathways among groups and to identify pathways associated with AP or T2DM.

### Statistical analysis

Nonparametric multivariate analysis of microbial community changes was performed using Mothur with default parameters to determine if within-state similarities in microbiome were significantly different from between-state similarities. The abundance of each species between the two groups was statistically compared using a T-test. A P-value of less than 0.05 was considered statistically significant.

## Results

### Subject characteristics

A total of 18 participants undergoing root canal treatment were enrolled in this study (**Table 1**). We collected 10 root canal samples from patients with AP (designated as AP) and 8 root canal samples from individuals with AP complicated by T2DM (designated as APDM). Prior to sample collection, participants underwent a comprehensive oral evaluation, and their HbA1c levels were assessed by clinicians at the outpatient clinic of the Department of Stomatology, Guang’an People’s Hospital. Significant differences in HbA1c levels were observed between the AP and APDM groups (P<0.001). Participant characteristics are detailed in **Table 1**.

**Table 1:** Characteristics of the study population.

Characteristics	AP	APDM
	(n=10)	(n=8)
Age (years)	63.59.3	64.0 ± 8.3
Gender (male/female)	6/4	4/4
Location (maxilla/mandibular)	5/5	4/4
HbA1c (%)	5.15 ± 0.44	7.67 ± 0.59*
BMI (kg/m^2^)	26.45 ± 1.95	27.35 ± 1.23

AP, Apical periodontitis; APDM, AP complicated with T2DM. *Statistically significant.

### 16S rDNA sequencing and coverage

Eighteen samples underwent 16S rDNA sequencing, all of which successfully passed quality control. The total number of valid reads from the 16S rDNA sequencing was 1,046,928, with a mean of 58,162 reads per sample. Utilizing a 97% similarity threshold, a total of 7,621 operational taxonomic units (OTUs) were identified.

**Figure 1:**
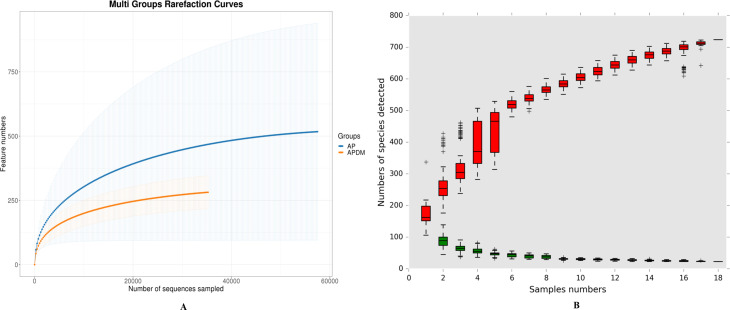
Coverage analysis among AP, and APDM. **(A)** Rarefaction curves reflects the intensity of the sequencing volume. **(B)** Cumulative curve of relative abundance of species reflects the relationship between the number of samples and the number of species noted.

We analyzed the alpha diversity of each sample, with the rarefaction curve, cumulative curve, Chao1 index, and Shannon index depicted in **Figure 1**, illustrating the relationship between bacterial abundance and diversity, evenness in infected root canal samples, or sampling intensity. The results indicated that feature discovery in all samples was saturated and comprehensively captured by the sequencing analysis in this study, as evidenced by the rarefaction curves (**Figure 1A**). The cumulative curve, reflecting the relationship between the number of samples and the number of species, suggested that both the sample size and species richness in this study were sufficient for data analysis, as they were nearing saturation (**Figure 1B**).

### Differences in microbial community structure between AP and APDM groups

**Figure 2:**
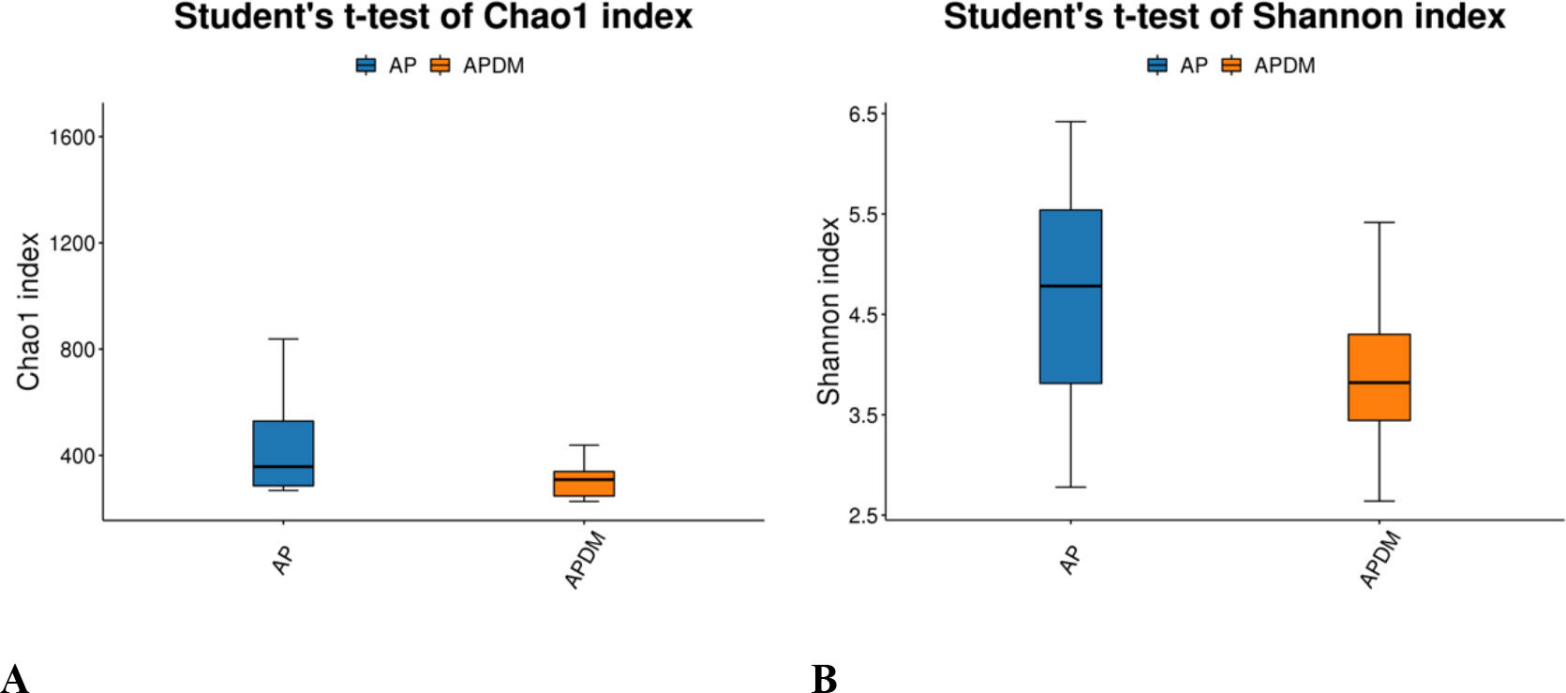
Alpha diversity analysis among AP, and APDM. **(A)** Chao1 index reflects bacterial community richness. **(B)** Shannon index reflects bacterial community diversity.

Alpha diversity analysis, encompassing community richness (Chao1 index) and community evenness (Shannon index), revealed no significant differences between the two groups (**Figures 2A, B**), suggesting similar species richness and diversity across groups.

**Figure 3:**
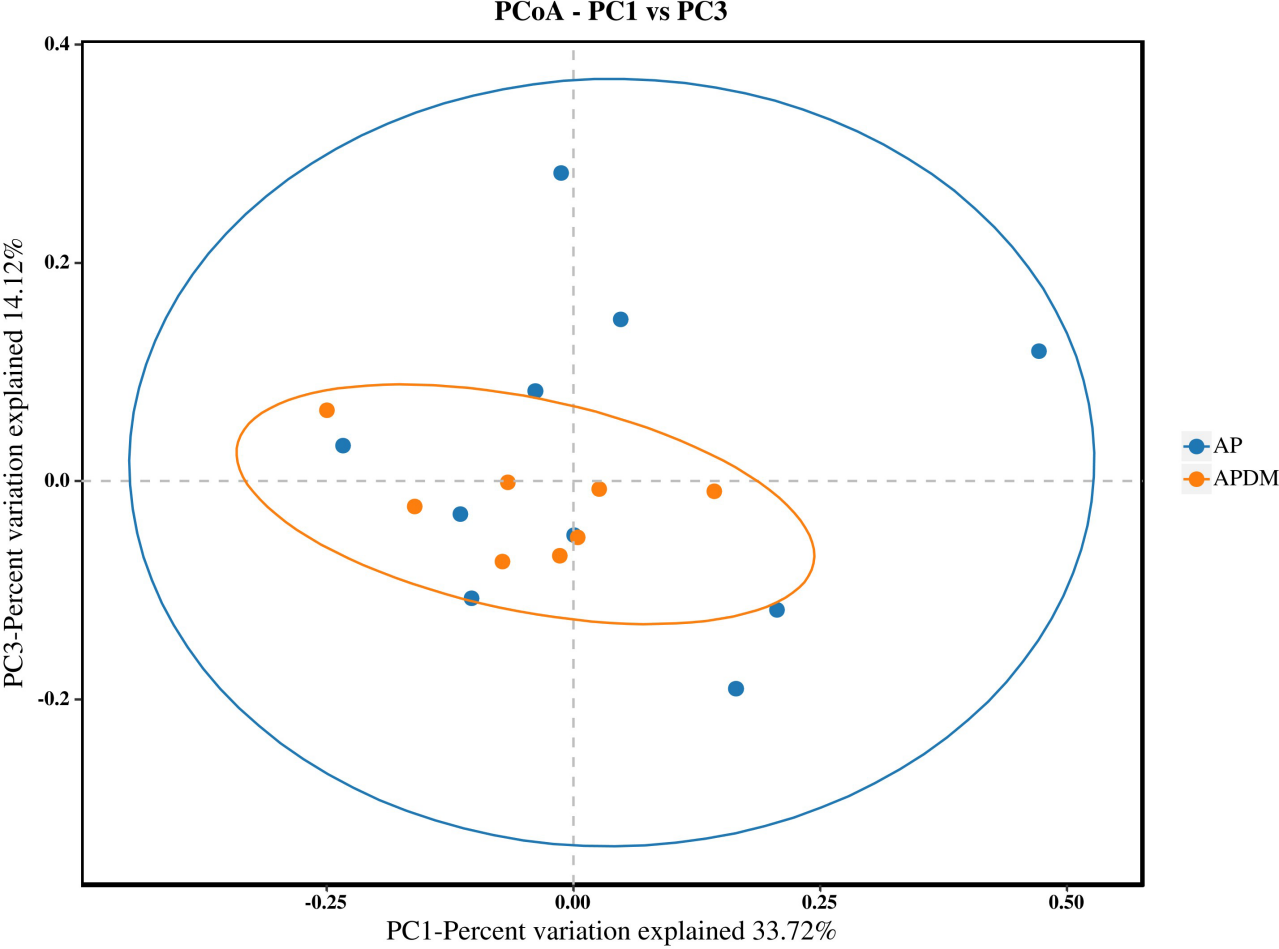
Beta diversity analysis among AP, and APDM. The plot of PCoA shows intergroup distances by 2 principal coordinates.

Beta diversity analysis was conducted to illustrate divergence between the AP and APDM groups. The principal coordinates analysis (PCoA) plot indicated similar species diversity and taxonomic compositions among the microbial communities of the two groups (Anosim analysis, P=0.181) (**Figure 3**).

**Figure 4:**
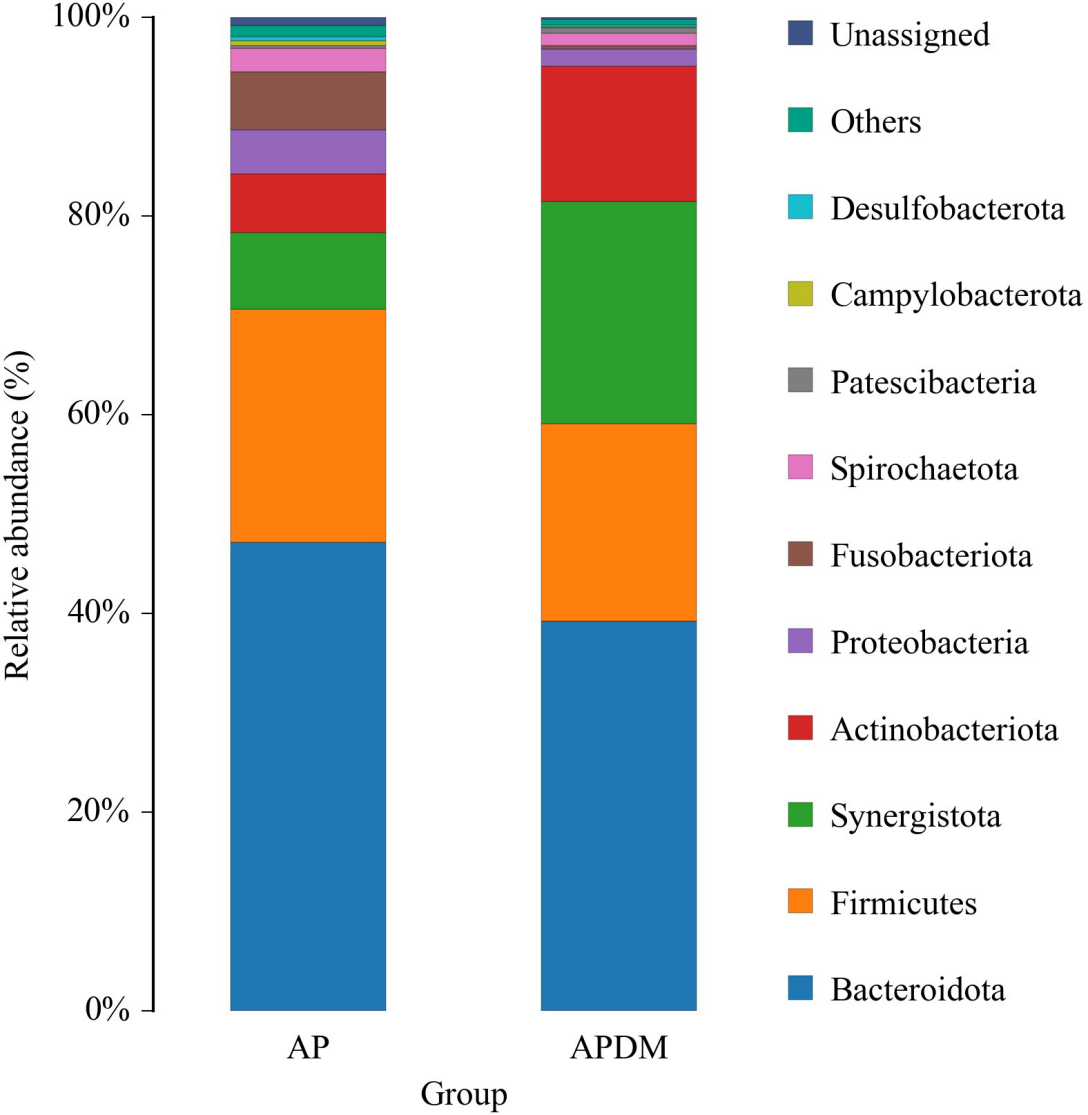
The relative abundance of bacterial taxa at the phylum level of AP, and APDM. Only the top ten species in the abundance level are displayed, and other OTUs are combined into Others and displayed in the figure.

The distribution of the top 10 phyla is shown in **Figure 4**. The core microbiota of root canal microorganisms in both the AP and APDM groups included Bacteroidota, Firmicutes, Synergistota, Actinobacteriota, Proteobacteria, Fusobacteriota, Spirochaetota, Patescibacteria, Campylobacterota, and Desulfobacterota. Compared to the AP group, the APDM group showed a significant reduction in Fusobacteriota and Campylobacterota, while Synergistota were significantly more abundant.

**Figure 5:**
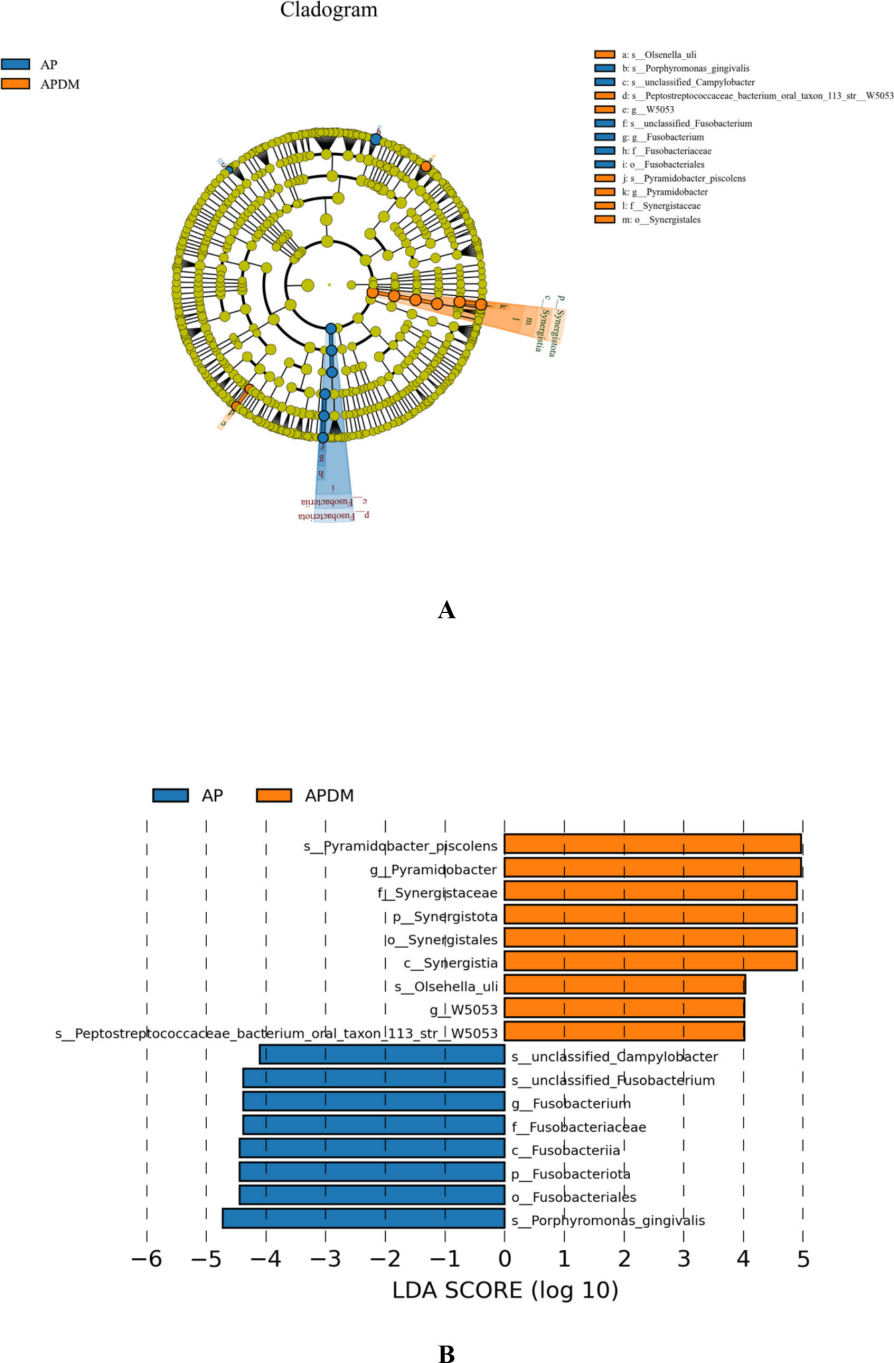
The linear discriminant analysis (LDA) effect size (LEfSe) profiles of AP, and APDM. **(A)** Cladograms indicating the phylogenetic distribution of bacterial lineages among 2 groups. The phylum, class, order, family, and genus levels are listed in order from inside to outside of the cladogram, and the labels for levels of order, family, and genus are abbreviated by a single letter. **(B)** LDA along with effect size measurements was applied to present the enriched bacterial genera of each group.

Linear discriminant analysis (LDA) effect size (LEfSe) analysis was employed to identify species with significantly different abundances between the two groups. As shown in **Figure 5**, a total of 9 genera (represented by Pyramidobacter_piscolen, Synergistaceae, and Olsenella_uli) were significantly more abundant in the APDM group, while 8 genera (represented by Porphyromonas_gingivalis, Fusobacterium, and unclassified_Campylobacter) were more abundant in the AP group. These 17 species, exhibiting significant differences, could potentially serve as biomarkers to distinguish between the AP and APDM groups (LDA>2, P<0.05).

### KEGG functional enrichment prediction of microbial community

**Figure 6:**
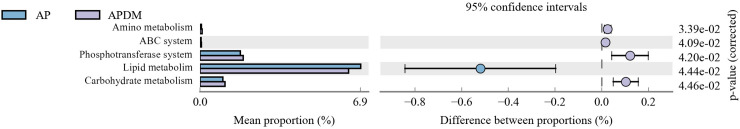
The histogram of Kyoto Encyclopedia of Genes and Genomes (KEGG) pathway categories based on differently detected genera of AP, and APDM.

Multiple KEGG pathways were significantly enriched in the disease states. The top 5 pathways enriched by the differentially detected microbial community are presented in **Figure 6** (P <0.05). Compared with the AP group, the APDM group showed enrichment in Phosphotransferase system, ABC system, Amino acid metabolism, and Carbohydrate metabolism pathways, while the AP group exhibited enrichment in the Lipid metabolism pathway.

## Discussion

The high prevalence of type 2 diabetes mellitus (T2DM) positions it as one of the most common chronic systemic diseases encountered in dental patients (Niazi and Bakhsh, 2022). Interactions between apical periodontitis and T2DM are likely influenced by a multitude of factors, including microbiota, host immune responses, oxidative stress, and more (Jain et al., 2019; Barcelos et al., 2020). Despite this, there remains a dearth of systematic and robust studies examining the relationship between these two conditions globally. Imbalances in microbial communities, associated with shifts in environmental conditions, suggest that microorganisms may significantly contribute to the progression of apical periodontitis in individuals with T2DM.

Our study’s findings indicate that the Chao1 and Shannon indices were lower in the APDM group compared to the AP group, suggesting a trend towards reduced bacterial richness and diversity within the infected root canals of teeth affected by APDM. Although not statistically significant, this trend implies that T2DM exerts some influence on the microbial community within the infected root canal of teeth with apical periodontitis. This observation is consistent with Goodson et al.’s research (Max Goodson et al., 2017), which reported a reduction in 35 out of 42 microbial species in the saliva of T2DM patients compared to healthy controls. Such a reduction could be linked to metabolic dysregulation and heightened immune responses in T2DM patients. Further PCoA analysis revealed similarities in the community structure between the AP and APDM groups, with a stronger clustering tendency observed in the APDM group and greater dispersion in the AP group. This finding corroborates previous research suggesting that T2DM does not significantly alter the subgingival microbial community structure in periodontitis (Balmasova et al., 2021), indicating a similar trend in apical periodontitis.

Regarding floral composition, the APDM group exhibited higher, though not significantly so, abundance of Actinobacteriota and Synergistota compared to the AP group. Long et al.’s prospective cohort study (Long et al., 2017) identified a significant correlation between Actinobacteriota and diabetes mellitus, potentially due to Actinobacteriota’s production of N-acetyl-β-D-glucosaminidase (NAG), which is prevalent in the saliva of T2DM patients and implicated in glycemic control. The higher, albeit not statistically significant, abundance of Actinobacteriota in the APDM group may be attributed to various factors, including case selection, sample size, and sampling methodology. T2DM patients often experience impaired hypothalamic insulin signaling, leading to decreased Branched-chain amino acids (BCAAs) breakdown and elevated circulating BCAAs levels (Wang et al., 2021). Synergistota, known for its conditional pathogenicity and synergistic role in microbial infections through amino acid breakdown, may also play a part (Manoharan et al., 2020).

Analysis of species with significant differences between the AP and APDM groups revealed that Olsenella, Peptostreptococcus, and Pyramidobacter were more abundant in the APDM group. Peptostreptococcus, linked to carbohydrate breakdown and acid resistance, may contribute to the higher incidence of dental caries in T2DM patients (de Brito et al., 2020; AL-Janabi, 2023). Olsenella has been identified as a potential causative agent of periapical infections, associated with abscess and fistula formation (Buysschaert et al., 2020), while Pyramidobacter, part of the Synergistota, can synergize with other microorganisms to cause infections under changed environmental conditions (Tzanetakis et al., 2015). These species could potentially serve as biomarkers for apical periodontitis complicated by T2DM.

Our detailed analysis of the APDM group’s root canal microbial community revealed significant enrichment in KEGG functional pathways, particularly the Phosphotransferase system, ABC system, Carbohydrate metabolism, and Amino acid metabolism pathways. The Phosphotransferase system, primary for microbial glucose transport, may be upregulated due to increased glucose content in the root canal microenvironment of APDM patients, promoting pathogenic microbial glucose transport activities (Zeng et al., 2023). The ABC system, essential for microbial nutrient acquisition, enhances glucose transport and metabolism, converting glucose into pyruvic acid, butyric acid, and other compounds that are further metabolized into short-chain fatty acids (SCFAs) [Kim et al., 2023. Sakanaka et al (Sakanaka et al., 2017). found higher levels of pyruvic acid and butyric acid in the gingival sulcus fluid of periodontitis patients, inducing inflammation and apoptosis in human periodontal ligament cells (hPDLCs), with butyric acid reducing insulin sensitivity and affecting glucose regulation (Gao et al., 2009). These findings suggest that the APDM group’s root canal microbiota may possess an enhanced metabolic capacity to adapt to intracanal microenvironmental changes.

Previous studies on the oral flora of T2DM patients have focused on various microenvironments, such as saliva and gingival crevicular fluid, using diverse methodologies and yielding varied perspectives. Our study utilized 16S rDNA sequencing to investigate root canal microorganisms in AP and APDM patient groups. However, limitations exist; while most microorganisms were detected, some remained unidentified using the V3-V4 hypervariable region, possibly including T2DM-associated pathogens. A recent study (de la Torre-Luna et al., 2019) reported a significant increase in Candida albicans in infected root canals of T2DM patients, associated with apical periodontitis prevalence, which our technique could not detect. Future studies may benefit from metagenomic sequencing for a comprehensive understanding of the root canal microbial community. Additionally, while our sample size aligns with other microbial sequencing studies, larger studies are warranted to validate our findings.

## Data Availability

The original contributions presented in the study are included in the article/supplementary material. Further inquiries can be directed to the corresponding author.
